# Plasma concentrations of dasatinib have a clinical impact on the frequency of dasatinib dose reduction and interruption in chronic myeloid leukemia: an analysis of the DARIA 01 study

**DOI:** 10.1007/s10147-018-1300-9

**Published:** 2018-05-29

**Authors:** Shuichi Mizuta, Masashi Sawa, Hisashi Tsurumi, Kana Matsumoto, Kotaro Miyao, Takeshi Hara, Takeshi Takahashi, Reona Sakemura, Hiroshi Kojima, Akio Kohno, Mari S. Oba, Satoshi Morita, Junichi Sakamoto, Nobuhiko Emi

**Affiliations:** 1 Department of Hematology, Fujita Health University School of Medicine, Toyoake, Japan; 2 Department of Hematology and Oncology, Anjo Kosei Hospital, Anjo, Japan; 3 Department of Hematology, Graduate School of Medicine, Gifu University, Gifu, Japan; 4 Department of Clinical Pharmaceutics, Doshisha Women’s College of Liberal Arts, Kyoto, Japan; 5 Department of Hematology, Gifu Municipal Hospital, Gifu, Japan; 6 Department of Hematology, Daido Hospital, Nagoya, Japan; 7 Department of Hematology and Oncology, JA Aichi Konan Kosei Hospital, Konan, Japan; 8 Department of Medical Statistics, Faculty of Medicine, Toho University, Tokyo, Japan; 9 Department of Biomedical Statistics and Bioinformatics, Kyoto University Graduate School of Medicine, Kyoto, Japan; 10 Tokai Central Hospital, Kagamigahara, Japan; 11 Hematology and Immunology, Kanazawa Medical University, 1-1 Daigaku, Uchinada, Kahoku-gun, Ishikawa, 920-0293 Japan

**Keywords:** Dasatinib, Plasma concentration, Chronic myeloid leukemia, Treatment adherence, Individualized dasatinib therapy

## Abstract

**Background:**

Dasatinib has shown promising anti-leukemic activity against chronic myeloid leukemia (CML). However, patients receiving dasatinib frequently require dose reductions and treatment interruptions (treatment alteration).

**Methods:**

We prospectively analyzed the frequency and significance of treatment alteration during dasatinib therapy in patients with CML. In all patients, trough plasma concentrations of dasatinib (*C*_min_) at steady state were assessed on day 28 of therapy.

**Results:**

28% of patients had their doses reduced at a median of 42 days, and 25% of patients had temporarily interrupted at a median of 54 days after treatment initiation. The overall dasatinib treatment alteration-free rate at 1 year was 66%. Age was significantly correlated with *C*_min_ on day 28 (*p* = 0.014), and the correlation remained significant after adjusting dasatinib dose (g), body weight (kg) (*C*_min_/*D*/*W*) (*p* = 0.026). In the univariate analysis, deep molecular response, advanced PS, higher *C*_min_/*D*/*W* were associated with a significantly higher risk of treatment alteration (HR 4.19, 95% CI: 1.06–16.60, *p* = 0.041; HR 5.26, 95% CI: 1.33–20.80, *p* = 0.018; and HR 10.15, 95% CI: 2.55–40.48, *p* = 0.001, respectively). In the multivariate analysis, advanced PS and higher *C*_min_/*D*/*W* were correlated with the incidence of treatment alteration (HR 4.78, 95% CI: 1.01–22.70, *p* = 0.049; HR 6.17, 95% CI: 1.17–32.50, respectively).

**Conclusion:**

Current data demonstrate that patients treated with dasatinib who displayed a high *C*_min_/*D*/*W* value and/or advanced PS were at a high risk for altered treatment.

## Introduction

Dasatinib is a novel tyrosine kinase inhibitor (TKI) of *BCR-ABL* and *SRC* family kinases that has shown promising therapeutic effects in patients with chronic myeloid leukemia (CML) [1–6]. In the DASISION study, the cumulative rates of major molecular response (MMR) and molecular response at 5 years were significantly greater in patients treated with dasatinib compared with imatinib [4–6]. However, there were no significant differences in progression-free survival and overall survival (OS) at 5 years [6]. In the DASISION study, 39% of patients initially treated with dasatinib and 37% of patients treated with imatinib are no longer receiving their respective initial therapy, and this discontinuation might have limited the potential benefits of treatment [6]. With regard to the imatinib therapy, poor adherence seemed to be the predominant reason underlying the lack of adequate clinical responses [7]. Larson et al. focused on the imatinib pharmacokinetics and reported that plasma trough levels of imatinib at steady state (day 29) was a significant prognostic indicator of midterm and long-term clinical responses in CML patients [8]. Then, we prospectively analyzed the frequency and significance of dose reductions and treatment interruptions during dasatinib therapy (treatment alteration) in patients with CML focusing on trough plasma concentrations of dasatinib at steady state.

## Materials and methods

### Patients

We conducted a phase 2 study to evaluate continuity of dasatinib therapy in patients with chronic phase CML (CML-CP) in the DARIA 01 study (UMIN000007345). Patients enrolled in the study were at least 16 years of age, and had Ph-positive CML-CP and primary or acquired hematologic resistance or intolerance to prior TKI therapy (imatinib and/or nilotinib). CML-CP was defined as < 15% blasts, < 20% basophils, < 30% blasts and promyelocytes, and platelets > 100 × 10^9^/L in peripheral blood samples, and no extramedullary involvement. Patients were considered TKI-intolerant if they had previously only tolerated TKI doses less than 400 mg/day or had discontinued TKI therapy due to toxicity potentially related to imatinib at a dose of 400 mg/day or less. Primary resistance to prior TKI therapy was defined as lack of complete hematologic response (CHR) after 3 months, lack of major cytogenetic response (MCyR) after 6 months, and lack of complete cytogenetic response (CCyR) after 12 months. The present study was approved by the Institutional Review Board of the Fujita Health University School of Medicine and conformed to the provisions of the 1964 Declaration of Helsinki and its later amendments or comparable ethical standards. All patients provided written, informed consent before participating in the study.

### Study design and treatment

The primary objective of this study was to identify factors influencing the frequency and significance of treatment alteration during dasatinib therapy at 12 months. The secondary objective was to evaluate the correlation between pharmacokinetic (PK) parameters and treatment alteration of dasatinib therapy, adverse events (AEs). Dasatinib was administered at an initial dose of 100 mg once daily. Dose interruption or reduction was permitted in cases of non-hematologic toxicity grade 2 or greater, hematologic toxicity grade 3 or greater, or upon request from a patient. After a dose interruption or reduction associated with toxicity, dasatinib could be re-administrated at a dose of 50–100 mg/day if the toxicity recovered to Grade 1 or 0. Dasatinib dose adjustment upon re-administration was determined by the treating physician. Treatment was continued until disease progression or intolerable toxicity. During the study, CML therapies other than dasatinib were prohibited; however, patients were permitted to receive hydroxyurea to control elevated WBC and/or platelets.

### Evaluations

Patients were seen once-weekly for the first 4 weeks and once-monthly for the following 11 months. Treatment efficacy was determined on the basis of hematologic assessments, bone marrow cytogenetics, and molecular responses in peripheral blood as assessed every 3 months. Patients who achieved CCyR were not required to undergo bone marrow cytogenetic analysis. AEs were continuously assessed throughout the study, and they were graded according to National Cancer Institute Common Terminology Criteria for Adverse Events (CTCAE), version 3.0. Safety assessments included AEs, hematologic and cardiac enzyme levels, biochemical parameters, urinalysis, electrocardiography, and physical examination. Chest X-rays were conducted at baseline and once-weekly for the first 4 weeks. Following the first 4 weeks of the study, chest X-rays were conducted as required for the detection or monitoring of pleural effusion. All patients underwent *BCR-ABL* mutational analysis at baseline. mRNA from peripheral blood cells was collected and analyzed for *BCR-ABL* gene mutations using denaturing high-performance liquid chromatography and sequencing. *BCR-ABL* transcripts were analyzed by Biomedical Laboratories (BML) (Tokyo, Japan) according to the international scale (IS) [9]. A deep molecular response (DMR) was defined as less than 0.01% of the International Scale (*BCR-ABL* IS), corresponding to a 4-log reduction from a standardized baseline value. Progression was defined as a doubling of white blood cell count, loss of CHR, increase in Ph-positive bone marrow metaphase cells, transformation to accelerated phase/blast phase, or death from any cause. All patients were told that their status of dasatinib administration would be monitored, and they were required to declare unused tablets to their physician.

### PK analysis

Trough plasma concentrations of dasatinib (*C*_min_) at steady state were assessed on day 28 of therapy. Blood samples were collected in heparinized tubes and immediately centrifuged. The separated plasma samples were stored in polypropylene tubes at − 30 °C until the PK analysis. Plasma concentration of dasatinib was measured using high-performance liquid chromatography coupled with electrospray mass spectrometry (HPLC–MS) as previously described, with some modifications [10]. The lower limit of quantitation of dasatinib by this method was 0.1 ng/mL and intra- and inter-day variabilities were within 5.0%.

### Data analysis and statistical methods

Dasatinib dose reduction-free and/or interruption (treatment alteration) -free rates were estimated using the Kaplan–Meier product limit method. Differences between groups were analyzed using the log-rank test, the Fisher exact test was used to calculate *p* values for incidences of responses and pleural effusion. Univariate and multivariate Cox regression analyses were used to evaluate the predictive value of various clinical variables on the risk of treatment alteration. The following variables were evaluated: age range (< 60 or ≥ 60), CML status at dasatinib initiation (CP, CCyR, or DMR), CML profile (newly diagnosed, prior TKI intolerance, or prior TKI resistance), performance status (PS) at diagnosis (0 or 1), *C*_min_ on day 28 (< 1.4 or ≥ 1.4 ng/mL), *C*_min_ on day 28 after adjusting dasatinib dose (g), body weight (kg) (*C*_min_/*D*/*W*). The relationship between *C*_min_, *C*_min_/*D*/*W*, and age were evaluated using Pearson’s correlation coefficient. All of the statistical analyses were conducted using STATA 12 software (STATA Corp., College Station, TX, USA).

## Results

### Patients

Between April 2012 and September 2013, 32 CML-CP patients were enrolled in the study. Patient characteristics are summarized in Table 1. The median age was  51 years (range 20–86). Twenty-three patients (75.0%) were treatment-naïve, and the remaining 9 patients (25.0%) had been switched to dasatinib from another TKI due to treatment intolerance or resistance. Dasatinib treatment was initiated a median of 1 month (range 0–109 months) after CML diagnosis. Upon the initiation of dasatinib, 26 patients were in the CP, 2 were in CCyR, and 4 were in DMR. No patients had *BCR-ABL* mutations at baseline.

**Table 1:** Patient demographics and characteristics at baseline

Median age, years (range)	51 (20–86)
Sex	
Male/female	21/11
ECOG performance status	
0	29
1	3
Disease history	
Newly diagnosed	23
Resistant/Intolerant	9
Previous therapy for CML	
Imatinib	5
Nilotinib	3
Other	1
None	23
Disease status	
CP	26
CCyR	2
DMR	4
*BCR–ABL* mutation status	
Positive/negative	0/32

*CP* chronic phase;  *CCyR* cytogenetic complete remission; *DMR* deep molecular remission

### Toxic effects

Grade 3 or 4 neutropenia and/or thrombocytopenia occurred in 9 (28%) and 5 patients (16%), respectively. Hematological AEs requiring dasatinib dose reduction and/or treatment interruption occurred in 5 patients (16%), and these AEs typically resolved within 3 months. Grade 1 pleural effusion (PE) occurred in 3 patients (9%), and grade 2 PE occurred in 5 patients (16%). PE was treated with diuretics in 3 patients (38%), steroid therapy and dasatinib dose reduction in 2 patients (25%), diuretics and dasatinib dose reduction in 2 patients (25%), and dasatinib dose reduction alone in 1 patient (13%). Complete clinical and radiological resolution of PE was achieved in 5 patients (62%), and clinical symptoms resolved in the remaining 3 patients (38%).

### Dasatinib interruption and dose reduction

**Fig. 1:**
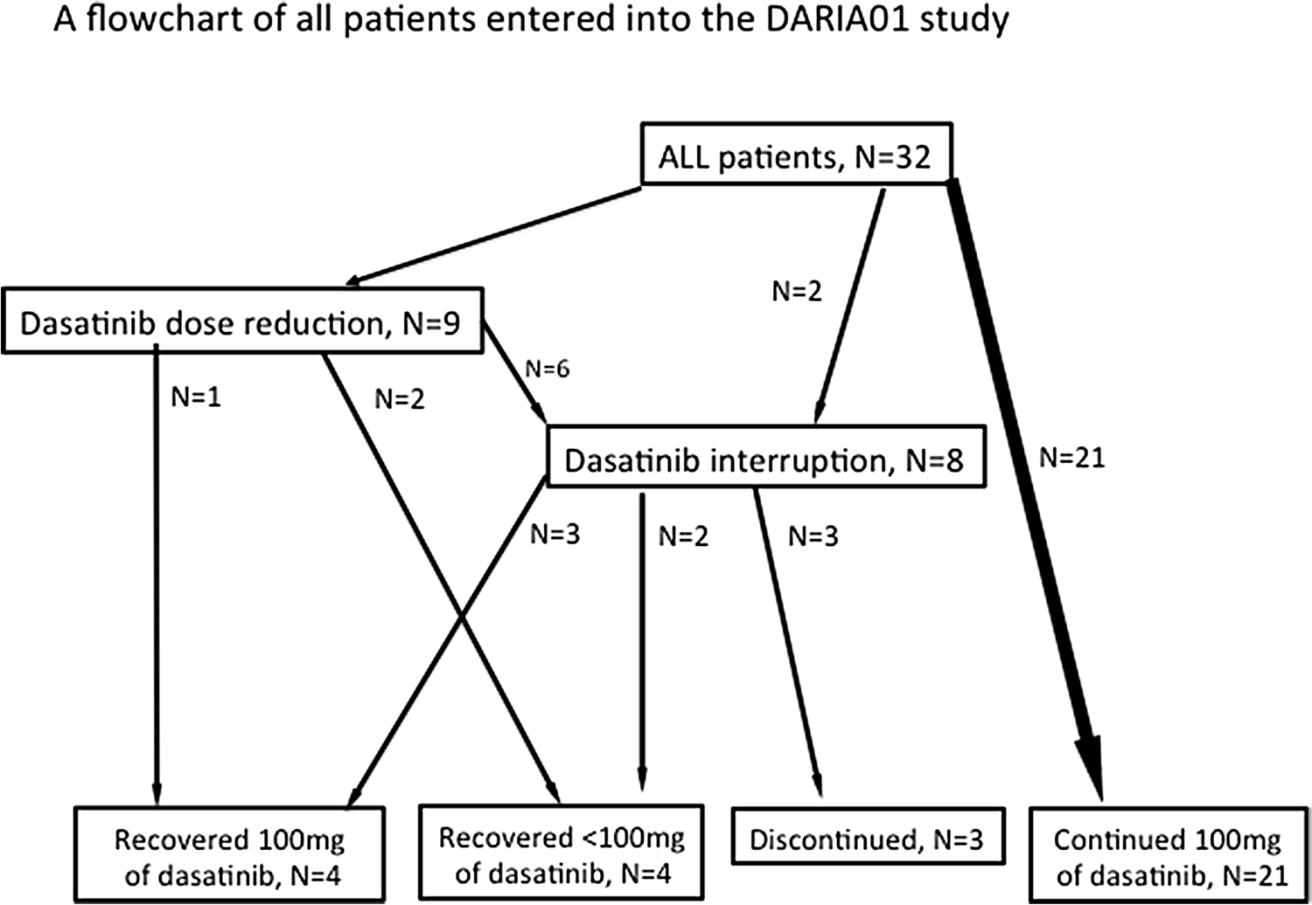
A flowchart of all patients entered into the DARIA 01 study

**Fig. 2:**
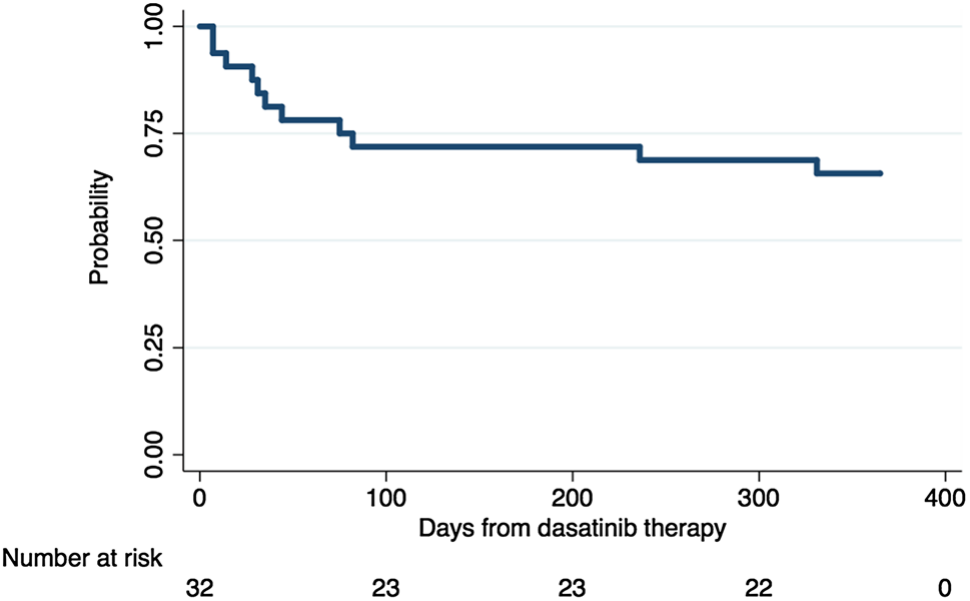
Dasatinib dose reduction- and/or interruption-free (alteration-free) rate

Figure 1 details the progress of all patients through the study. The dose of dasatinib was reduced in 9 patients (28%) at a median of 42 days (range 7–123 days) after treatment initiation. Treatment was temporarily interrupted in 8 patients (25%) at a median of 54 days (range 14–331 days) after treatment initiation. Among 8 patients who had PE, 5 patients experienced treatment alteration (reduction in 2 patients, reduction and interruption in 3 patients). Three patients (9%) discontinued treatment due to withdrawal of consent at 287 day, myelo-suppression at 236 days, or gastro-intestinal bleeding at 342 days. At the final observation, 25 patients were being treated with 100 mg of dasatinib, and 4 patients were being treated with a reduced dose (50 or 40 mg). The overall treatment alteration-free rate was 66% (95% confidence interval [CI]: 47–79%) (Fig. 2).

### Clinical response

The rates of CCyR and DMR in the 22 treatment-naïve patients were 68% and 4% at 3 months, 91% and 32% at 6 months, and 91% and 55% at 12 months, respectively. In the 10 patients who had been switched from other TKIs, 3 of the 4 patients in CHR achieved CCyR, and 1 of 2 patients in CCyR achieved a DMR at 3 months. The rates of CCyR and DMR were 90% and 60% at 3 months, 90% and 60% at 6 months, and 90% and 78% at 12 months, respectively. Twenty-nine of the 32 patients remained on dasatinib therapy at 1 year. No patients experienced disease progression while being treated with dasatinib. There was no significant difference in clinical responses in patients that underwent treatment alteration (*p* = 0.157) compared with patients that did not undergo these adjustments.

### Correlation of dasatinib trough levels with age

**Fig. 3:**
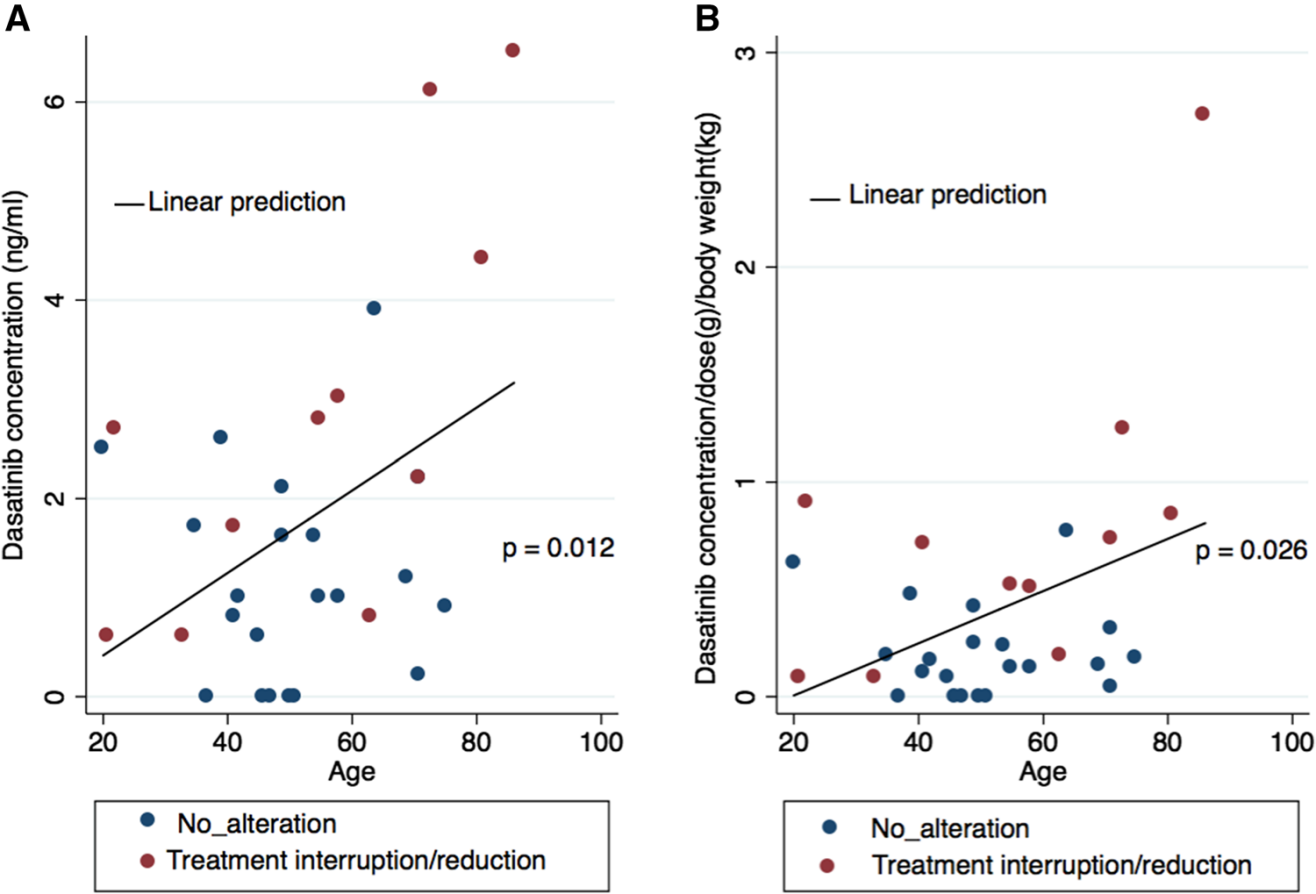
Relationship between age and dasatinib trough plasma concentrations

PK data associated with dasatinib on day 28 were available for all of the study participants. On day 28, 27 patients were still being treated with the initial dose of 100 mg, and the remaining 5 were being treated with a lower dose (50 mg) due to an AE. The median *C*_min_ of dasatinib was 1.4 ng/mL (range 0.0–6.0 ng/mL). The median *C*_min_ after adjusting for dasatinib dose (g) and body weight (kg) (*C*_min_/*D*/*W*), on day 28 was 0.19 (range 0.00–2.7). The inter-patient variability might reflect individual differences in drug metabolism and/or excretion. A linear regression analysis demonstrated that age was significantly correlated with *C*_min_ on day 28 (*r* = 0.431, *p* = 0.014; Fig. 3), and the correlation remained significant after adjusting for *C*_min_/*D*/*W* (*r* = 0.394, *p* = 0.026; Fig. 3).

### PE and dasatinib serum concentration

Among 16 patients who showed high *C*_min_ (≥ 1.4 ng/mL), five (31%) experienced PE. On the other hand, in 16 patients who showed low *C*_min_ (< 1.4 ng/mL), 3 (19%) experienced PE. There was no significance in the incidence of PE between two groups (*p* = 0.685). Among 16 patients who showed high *C*_min_/*D*/*W* (≥ 0.19), six (38%) experienced PE. On the other hand, in 16 patients who showed low *C*_min_/*D*/*W* (< 0.19), 2 (13%) experienced PE. The difference between two groups did not reach statistical difference (*p* = 0.220). The incidence of PE by 12 months in high age (≥ 60) group was higher than that in low age (< 60) group, but did not reach statistical significance (40% vs. 18%, *p* = 0.1864). With regard to adverse events other than PE, there were no significant correlation between AEs and dasatinib serum concentration. (*C*_min_, *C*_min_/*D*/*W*) (Supplemental tables 1, 2).

### Risk factors for dasatinib treatment alteration

**Fig. 4:**
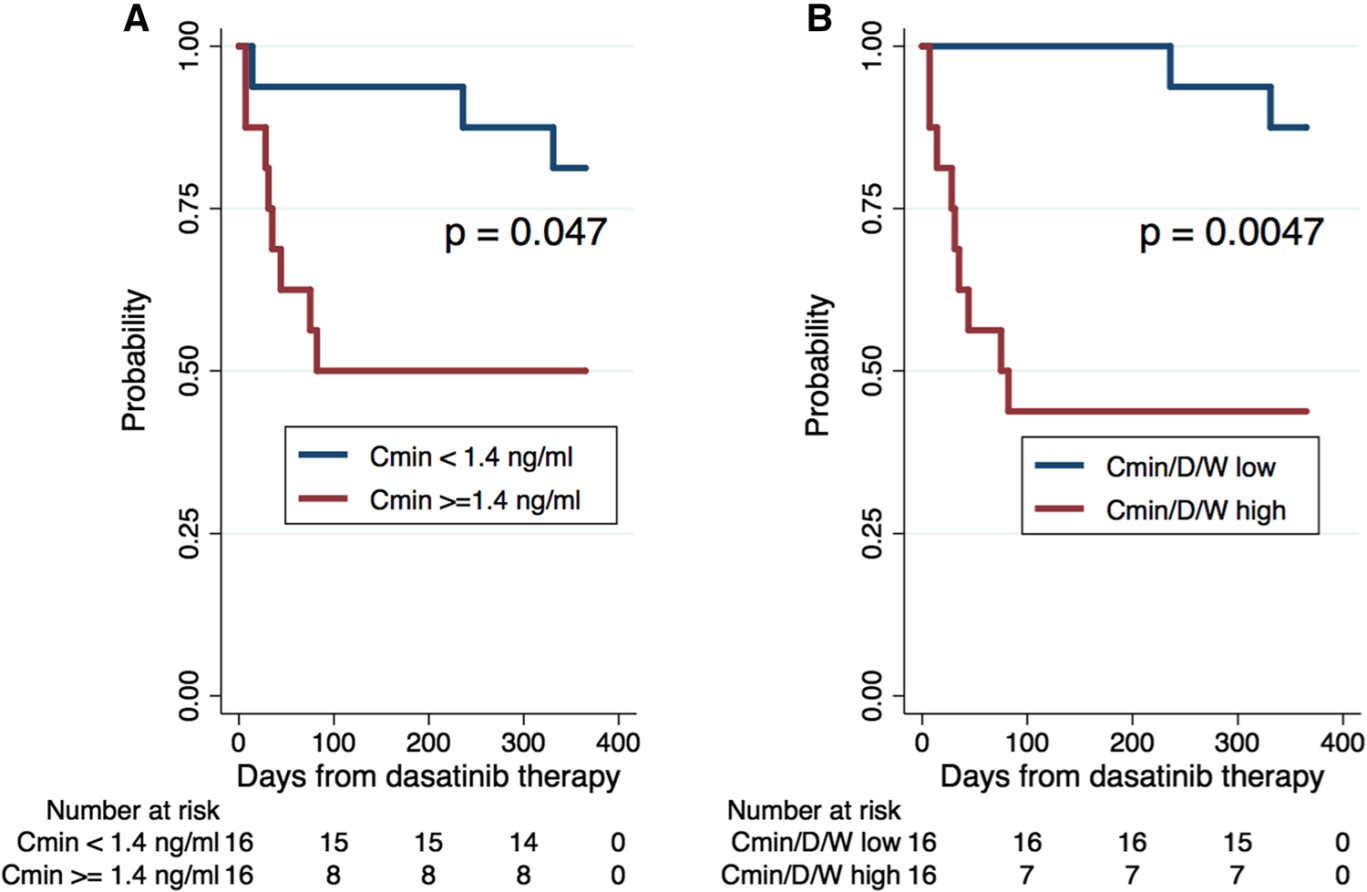
**a** 1-year dasatinib treatment alteration-free rate by dasatinib trough plasma concentrations (*C*_min_) (ng/mL) (1.4 ≧ vs. <1.4). **b** 1-year dasatinib treatment alteration-free rate by trough plasma concentrations of dasatinib after adjusting for dasatinib dose (g) and body weight (kg) (*C*_min_/*D*/*W* ratio) at day 28 (0.19 ≧ vs. < 0.19)

The treatment alteration-free rate was significantly greater in the low *C*_min_ group (< 1.4 ng/mL) (81, 95% CI: 52–94%) compared with the high *C*_min_ group (50, 95% CI: 25–71%, *p* = 0.0473) (Fig. 4). And also, the treatment alteration-free rate in low *C*_min_/*D*/*W* group (88, 95% CI: 59–97%) was significantly greater than that in the high *C*_min_/*D*/*W* group (44, 95% CI: 20–66%, *p* = 0.0047) (Fig. 4). Table 2 presents the results of the univariate and multivariate Cox regression analyses of risk factors for treatment alteration. In the univariate analysis, DMR, advanced PS, higher *C*_min_/*D*/*W* were associated with a significantly higher risk of treatment alteration (HR 4.19, 95% CI: 1.06–16.60, *p* = 0.041; HR 5.26, 95% CI: 1.33–20.80, *p* = 0.018; and HR 10.15, 95% CI: 2.55–40.48, *p* = 0.001, respectively). In the multivariate analysis, advanced PS and higher *C*_min_/*D*/*W* were still correlated with the incidence of treatment alteration (HR 4.78, 95% CI: 1.01–22.70, *p* = 0.049; HR 6.17, 95% CI: 1.17–32.50, *p* = 0.032).

**Table 2:** Relationship between the dasatinib interruption/reduction of treatment and the remaining variables

Characteristic	Univariate analysis		Multivariate analysis	
	HR (95% CI)	p	HR (95% CI)	*p*
Age				
> 60	1.00			
≥ 60	2.46 (0.75–8.14)	0.138	NA	
Sex				
Female	1.00			
Male	1.88 (0.57–6.17)	0.300	NA	
CML status at dasatinib initiation				
CP	1.00			
CyCR	2.52 (0.31–20.7)	0.388	1.26 (0.14–11.0)	0.834
DMR	4.19 (1.06–16.6)	0.041	1.21 (0.26–5.74)	0.809
Prior-therapy before dasatinib administration				
No	1.00			
Yes	2.60 (0.79–8.58)	0.116	NA	
PS at dasatinib treatment				
0	1.00	0.018		
1	5.26 (1.3–20.8)		4.78 (1.01–22.7)	0.049
*C*_min_ on day 28 (ng/mL)				
> 1.4	1.00			
≥ 1.4	3.53 (0.93–13.41)	0.063	NA	
*C*_min_/*D*/*W* on day 28				
> 0.19	1.00			
≥ 0.19	10.15 (2.55–40.48)	0.001	6.17 (1.17–32.5)	0.032

*CP* chronic phase; *CyCR* cytogenetical complete remission; *DMR* deep molecular remission; *C*_*min*_ plasma concentrations of dasatinib at steady state;  *C*_*min*_*/D/W,  C*_min_ after an adjustment in the dasatinib dose and body weight

## Discussion

It is now recognized that discontinuation of dasatinib therapy is a critical factor in the achievement and maintenance of an optimal response to dasatinib therapy [11]. In the DASISION study, dasatinib treatment was interrupted in 63% of patients, the dose of dasatinib was reduced in 31% of patients [5]. At the end of the 3-year observation period, 29% patients discontinued dasatinib therapy for various reasons (AE: 10%, disease progression or treatment failure: 10%, unrelated AE: 2%, death: 2%, or other reason: 5%) [5]. However, the detailed course leading to discontinuation in each case has not been reported. To the best of our knowledge, there have been no previous reports regarding the timing of dasatinib reduction, interruption, and/or discontinuation in midterm observation. In the present study, 28% of patients experienced a dose reduction and 25% of patients experienced temporarily interruption of treatment after initiating dasatinib therapy. The 1-year overall dasatinib treatment alteration-free rate was 66%. 9% of patients discontinued the treatment due to withdrawal of consent or an AE at 1-year from dasatinib therapy. No patients experienced disease progression while being treated with dasatinib.

As it often necessitates treatment discontinuation, PE can limit the therapeutic efficacy of dasatinib [12–14]. On the other hand, several studies reported that the pleural effusion was common in elderly patients and did not affect any negative treatment results if that were clinically manageable [15, 16]. In the present study, the incidence of PE by 12 months in high age (≥ 60) group was higher than that in low age (< 60) group, but did not reach statistical significance (*p* = 0.1864). Several studies have reported that a higher Cmax of dasatinib was associated with greater clinical response rates, and lower trough concentrations were associated with a lower risk of PE [12, 17–19]. In the prospective OPTIM dasatinib trial, patients with a dasatinib trough concentration of − 3 nM (1.5 ng/mL) at day 15 were randomized to the non-dose adjustment group or the dose adjustment group to obtain a C0 of < 3 nM [20]. The overall rates of pleural effusion at 36 months were 49% and 11%, respectively, in the non-adjustment and adjustment groups, and the discontinuation rates of dasatinib therapy were 27% and 13%, respectively (*p* = 0.008) [20]. They concluded that monitoring dasatinib PK parameters could help predict the risk of side effects and prevent discontinuation of dasatinib therapy [20]. In the present study, patients who had high *C*_min_/*D*/*W* showed higher incidence of PE than that in low *C*_min_/*D*/*W* group (38% vs. 13%, respectively), despite it did not reach statistical significance (*p* = 0.220). Indeed, among 8 patients who had PE, 5 patients experienced treatment alteration. However, pleural effusions were well managed effectively through administration of diuretics or steroid therapy and/or dasatinib dose modifications. After then, they could continue dasatinib therapy. Better management of PE might contribute to prevent discontinuation of dasatinib therapy. In the present study, age was significantly correlated with *C*_min_ (*p* = 0.012) (Fig. 2), and the correlation remained significant after adjusting for *C*_min_/*D*/*W* (*p* = 0.026). The treatment alteration-free rate was significantly greater in the low *C*_min_ group (< 1.4 ng/mL) compared with the high *C*_min_ group (81% and 50%, respectively, *p* = 0.0473) (Fig. 4). And also, the treatment alteration-free rate in low *C*_min_/*D*/*W* group was significantly greater than that in the high *C*_min_/*D*/*W* group (88% and 44%, respectively, *p* = 0.0047) (Fig. 4). The higher *C*_min_/*D*/*W*, instead of higher *C*_min_, was significantly correlated with the incidence dasatinib treatment alteration rate in the multivariate analysis. The *C*_min_/*D*/*W* possibly could reflect patient’s ability to metabolize and/or excretion dasatinib. A larger patient sample is needed to determine which would be better to predict the dasatinib treatment alteration and to prevent discontinuation of dasatinib therapy.

To our knowledge, this is the first prospective study evaluating prognostic factors that influence the treatment alteration of dasatinib therapy in patients with CML. However, the limitations of our study need to be considered. Our study was limited by the presence of residual confounding factors, both known and unknown.

The present study focused on clinical outcomes of 32 patients at the midterm point (1 year) of the observation period of the DARIA 01 study; however, dasatinib therapy can be continued for much longer periods of time. A long-term observation study including more large number of patients is needed to clarify the risks for the treatment alteration of dasatinib therapy.

## Conclusion

We demonstrated that trough plasma concentrations of dasatinib after adjusting for dasatinib dose and body weight (*C*_min_/*D*/*W*) at day 28 and performance status (PS) at diagnosis were risk factors for treatment alteration during the midterm point in the observation period of the DARIA 01 study. Monitoring dasatinib PK parameters could help to predict the risk of treatment alteration of dasatinib therapy and optimize the efficacy of dasatinib therapy.
